# An Examination of Personality Traits Associated with Autonomous Sensory Meridian Response (ASMR)

**DOI:** 10.3389/fpsyg.2017.00247

**Published:** 2017-02-23

**Authors:** Beverley Fredborg, Jim Clark, Stephen D. Smith

**Affiliations:** Department of Psychology, University of WinnipegWinnipeg, MB, Canada

**Keywords:** Autonomous Sensory Meridian Response (ASMR), Big Five Inventory, personality, neuroticism, openness

## Abstract

Autonomous Sensory Meridian Response (ASMR) is a perceptual condition in which the presentation of particular audio-visual stimuli triggers intense, pleasurable tingling sensations in the head and neck regions, which may spread to the periphery of the body. These triggering stimuli are often socially intimate in nature, and usually involve repetition of movements and/or sounds (e.g., hearing whispering, watching someone brush her hair). Reports of ASMR experiences first appeared in online communities in 2010; since this time, these communities have expanded, with some groups consisting of over 100,000 members. However, despite the apparent prevalence of ASMR, there is currently no research on the personality characteristics that co-occur with this condition. In the current study, 290 individuals with ASMR and 290 matched controls completed the Big Five Personality Inventory (BFI; John et al., 1991); participants with ASMR also completed a questionnaire related to their ASMR phenomenology. Individuals with ASMR demonstrated significantly higher scores on Openness-to-Experience and Neuroticism, and significantly lower levels of Conscientiousness, Extraversion, and Agreeableness compared to matched controls. Further, ratings of subjective ASMR intensity in response to 14 common ASMR stimuli were positively correlated with the Openness-to-Experience and Neuroticism dimensions of the BFI. These results provide preliminary evidence that ASMR is associated with specific personality traits and suggest avenues for further investigation.

## Introduction

Autonomous Sensory Meridian Response (ASMR) is a perceptual condition in which specific stimuli (ASMR “triggers”) reliably elicit relaxing and pleasurable tingling sensations that are initially localized to the head and neck region and may spread secondarily to other regions of the body. ASMR triggers vary from person to person and may be auditory, visual, tactile, and/or olfactory in nature.

ASMR was recently brought to the attention of the public in 2010 (del Campo and Kehle, 2016). At this time, numerous online forums included discussions of a previously-unnamed feeling termed Autonomous Sensory Meridian Response, an unscientific name coined by Jennifer Allen. Over the next few years, descriptions of ASMR proliferated in the media, with some journalists referring to the tingling phenomenology as “brain orgasms” (e.g., Beck, 2013). To date, little research has been published on the phenomenon; indeed, only five peer-reviewed papers have been published on ASMR (e.g., Ahuja, 2013; Andersen, 2015; Barratt and Davis, 2015; del Campo and Kehle, 2016; Smith et al., 2016), only two of which included empirical data Barratt and Davis, 2015; Smith et al., 2016). Due to a dearth of experimental research on the subject, our understanding of the formal descriptive parameters of ASMR is highly limited. A recent survey study by Barratt and Davis (2015) was the first to find that whispering, close-up attention, and slow movements such as hair-brushing elicited tingles in over half of the 450 individuals with ASMR that they studied. The authors also found that other common ASMR triggers include listening to and watching an individual tap on various objects, watching an individual open a package, and watching an individual complete an intricate task, such as drawing, painting their fingernails, or applying make-up.

There are several factors that distinguish ASMR from other atypical sensory experiences, such as *frisson*—the pleasurable tingling sensations that occur during an emotional response to music (often referred to as “chills”; del Campo and Kehle, 2016). The two phenomena are similar in that they both tend to occur while one is mindful and fully engaged with the triggering stimulus, they both involve an affective component, and both experiences are associated with large individual differences in triggering stimuli (Nusbaum et al., 2014; del Campo and Kehle, 2016). However, the two phenomena differ in that the tingles associated with frisson tend to spread rapidly throughout the body, whereas ASMR-associated tingles may last upwards of several minutes (del Campo and Kehle, 2016). Further, unlike frisson, ASMR experiences are often described as “wave-like” and “dynamic,” as the intensity of the tingles tends to morph throughout the triggering experience and may spread from the head and neck regions to the periphery of the body (Barratt and Davis, 2015; del Campo and Kehle, 2016). Finally, and perhaps most importantly for those who experience both ASMR and *frisson*, the tingling sensations associated with ASMR are often associated with relaxation and contentment (Barratt and Davis, 2015), whereas *frisson* experiences may be due to an exciting or emotionally arousing experience (del Campo and Kehle, 2016).

Consistent sensory associations *do* occur in synesthesia, a perceptual condition that shares some characteristics with ASMR. Individuals with synesthesia experience otherwise unrelated secondary sensations to specific sensory stimuli (Cytwowic, 1989; Hubbard and Ramachandran, 2005). For instance, people with synesthesia may experience a specific taste when they hear a particular sound or see color photisms when viewing numbers or letters. In both ASMR and synesthesia, the same perceptual or cognitive stimuli tend to reliably and automatically elicit the same atypical sensory response (e.g., synesthetic photisms or ASMR tingles). Both conditions are also associated with altered patterns of functional connectivity between brain regions, suggesting a possible neural mechanism for these experiences (Dovern et al., 2012; Smith et al., 2016). Interestingly, Barratt and Davis (2015) found in their survey that 5.9% of the ASMR population reported synesthetic experiences, suggesting a possible overlap between the two phenomena. However, ASMR and synesthesia differ in that the secondary sensory experiences associated with synesthesia are automatic and uncontrollable, whereas ASMR experiences are autonomous but can ostensibly be stopped by intentionally choosing to disengage from the triggering stimulus.

The fact that ASMR has phenomenological characteristics that differentiate it from experiences such as frisson and synesthesia suggests that this condition is a valid topic of scientific inquiry. The challenge for researchers is to identify the different factors that underlie ASMR. In previous research, we identified atypical patterns of functional connectivity as one potential causal factor for this condition (Smith et al., 2016). In the current study, we examined whether personality traits play a role in ASMR. Specifically, we investigated whether individuals with ASMR differed from matched control participants on five broad personality domains: Openness-to-Experience, Conscientiousness, Extraversion, Agreeableness, and Neuroticism (e.g., Costa and McCrae, 1992; John and Srivastava, 1999). Extrapolating from surveys of individuals with ASMR as well as from examinations of similar conditions, we predicted that individuals with ASMR would differ from matched controls on the personality dimensions of Openness-to-Experience and Neuroticism.

Openness-to-Experience is associated with curiosity, unconventionality, artistic or aesthetic tendencies, wide interests, and fantasy (John and Srivastava, 1999). People open to experience are generally curious about the world around them and may be prone to vivid fantasies or daydreams. In a study using the Revised NEO Personality Inventory (NEO-PI-R; Costa and McCrae, 1992), McCrae (2007) found that the best predictor of scores on the Openness-to-Experience dimension was the tendency to experience “chills” during aesthetic experiences, such as listening to music. These findings suggest that individuals with ASMR should score higher than matched controls on Openness-to-Experience, as the sensations associated with ASMR may be due, in part, to increased receptivity and sensitivity to sensation.

Neuroticism is associated with anxiety, angry hostility, depression, and self-consciousness (John and Srivastava, 1999). In a survey of people with ASMR, a significant proportion of individuals who demonstrated moderate to severe depression reported using ASMR-triggering stimuli to temporarily attenuate symptoms of depression and/or anxiety (Barratt and Davis, 2015). As a statistically significant proportion of Barratt and Davis (2015) participants experienced higher-than-normal levels of depression, we hypothesized that individuals with ASMR would similarly report higher scores on trait Neuroticism than matched controls.

The paucity of research into personality factors underlying atypical perceptual experiences limited our ability to make *a priori* predictions related to Conscientiousness, Extraversion, and Agreeableness. The current study will therefore provide novel insights into the relationship between these factors and phenomena such as ASMR.

In addition to examining the relationship between ASMR and personality traits *in general*, the current research also examined whether personality traits were linked to the observed heterogeneity in ASMR triggers and the intensity of individuals' ASMR experiences. To facilitate this investigation, an “ASMR checklist” was developed to assess various topics related to the condition, including the speed and intensity of ASMR responses to 16 stimuli that have been known to cause tingles in the ASMR population. We expected that those who regularly experienced high-intensity tingles would demonstrate higher Openness-to-Experience and Neuroticism scores as compared to those who report experiencing relatively low-intensity tingles. Together, these results will allow us to understand the constellation of personality traits associated with ASMR and whether these traits vary as a function of individuals' ASMR phenomenology.

## Methods and materials

### Participants

Participants were 290 adults with ASMR and 290 control group members matched for age and gender. Individuals who did not indicate a gender were excluded (6 ASMR participants and 5 Controls). Further, data from six individuals in the control group were removed as they had left comments in the feedback section suggesting that they intentionally distorted their responses. In total, 284 individuals with ASMR (*n* = 149 females, *M*_age_ = 28.07, *SD* = 9.58) and 279 control participants (*n* = 156 females, *M*_age_ = 29.19, *SD* = 10.55) were included in subsequent analyses.

Self-identifying ASMR participants were recruited for a research study on ASMR and personality traits via the popular forum website Reddit (http://www.reddit.com); Reddit contains forums, or *subreddits*, on many topics, including one dedicated to ASMR (http://www.reddit.com/r/ASMR). This recruitment strategy allowed us to survey a large and diverse group of individuals with ASMR, providing an ideal opportunity to examine the personality characteristics associated with this condition. A message on the website invited individuals who self-identified as having ASMR to complete a survey hosted on the survey website *Qualtrics* (Qualtrics, Inc., Provo, UT). Participants therefore volunteered at their own discretion. Qualifying questions related to ASMR were asked in order to validate participants' inclusion in the study.

Control participants were recruited via Qualtrics Panels. Members of the Qualtrics Panels team coordinated the recruitment of control participants based on their age and sex. To ensure that none of the control participants had ASMR without their knowledge, these participants viewed two online videos that typically elicit ASMR tingles prior to beginning the study. If a potential participant reported experiencing tingles, his or her data were not recorded.

All participants gave informed consent and the study was approved by the Research Ethics Committee of the Psychology Department at the University of Winnipeg.

### Materials

The on-line questionnaire included demographic questions, the Big Five Inventory (BFI; John et al., 1991), the Toronto Mindfulness Scale (Lau et al., 2006), the Mindful Attention and Awareness Scale (Brown and Ryan, 2003), a self-created Embodied Emotion Scale, and an ASMR checklist. The present report focuses on the BFI and a self-created ASMR checklist.

The BFI consisted of 44 items, with 8–10 items for each of the five scales. Reliabilities in the present study were excellent as measured by Cronbach's alpha: Openness-to-Experience (0.77), Conscientiousness (0.83), Extraversion (0.84), Agreeableness (0.80), and Neuroticism (0.83). Reliabilities were equivalent for the ASMR and Control groups.

The ASMR questionnaire was administered only to ASMR participants and consisted of a series of questions about whether participants experienced ASMR responses to 16 stimuli known to elicit tingles in the ASMR population (e.g., whispering, haircut simulations). The specific ASMR triggers included in the ASMR Checklist were selected based on interviews with individuals with ASMR and an examination of social media reports on internet sites such as reddit.com/ASMR and youtube.com; the ASMR Checklist was revised based on the results of the survey study by (Barratt and Davis, 2015). Individuals were asked to rate each stimulus on a 7-point scale from 0 to 6, with a rating of “0” representing “no tingles,” a rating of “3” representing “moderately intense tingles,” and a rating of “6” representing the “most intense ASMR experience.” “Unknown” was also an option and would be the appropriate response if, for example, participants had never been exposed to that particular stimulus or could not recall an experience with that stimulus. Participants also estimated approximately how long it would take for the tingles to be perceived after stimulus onset. Two of the 16 items were removed from the checklist due to a very high number of “unknown” responses (*N* > 100). Furthermore, neither of these stimuli were on the list of common ASMR-triggering stimuli by (Barratt and Davis, 2015). As such, we deemed it appropriate to remove these items from the checklist for the purposes of data analysis.

Following the ASMR stimulus evaluation questions, participants were asked a variety of questions about their ASMR experiences, such as how often ASMR videos were used to help them sleep or relax, if they experienced frisson, and how pleasurable ASMR experiences were. This section of the ASMR Checklist was constructed based on a review of online reports of ASMR triggers, and was confirmed by the list of common ASMR triggers outlined in the survey study by (Barratt and Davis, 2015). A text version of the checklist is included in Appendix A.

### Procedure

At the start of the study, potential participants indicated that they had read and accepted the conditions of an informed consent form. Respondents who did not agree to the study were guided to a termination screen and thanked for their time. Once consent was provided, participants completed the six different questionnaires. The order of the questionnaires was randomized for each participant. After the completion of all questionnaires, participants answered a series of standard demographic questions. Upon conclusion of the study, participants received an online feedback form thanking them for their time.

## Results

We first report comparisons between the ASMR and Control groups for the Big Five Inventory, followed by the psychometric properties of our novel ASMR checklist, and then a preliminary analysis of BFI results as a function of responses to the ASMR checklist.

### The big five inventory

As noted earlier, the BFI scales were highly reliable and scores for each domain were calculated according to a standard scoring protocol (John et al., 1991). These scores were analyzed separately with Group and Gender as factors. Gender was included due to the well-documented differences between males and females on several BFI scales (Costa et al., 2001; e.g., Weisberg et al., 2011). Interactions between Group and Gender are not discussed below except where Gender moderated completely the difference between ASMR and Control participants. The means and standard deviations for each scale are reported in Table 1 and analyzed below.

**Table 1:** **Mean big five inventory scores as function of group and gender**.

	**Females**				**Males**			
**BFI scale**	**Control *M***	**Control *SD***	**ASMR *M***	**ASMR *SD***	**Control *M***	**Control *SD***	**ASMR *M***	**ASMR *SD***
Openness to experience	3.445	0.5774	4.042	0.4823	3.444	0.5904	3.762	0.6048
Conscientiousness	3.711	0.6599	3.243	0.7403	3.583	0.7855	3.107	0.6581
Extraversion	3.057	0.8038	2.816	0.8483	3.145	0.7439	2.517	0.7941
Agreeableness	3.768	0.7001	3.652	0.6587	3.636	0.7847	3.428	0.6384
Neuroticism	3.112	0.7880	3.596	0.7732	2.801	0.8485	3.103	0.7699

As predicted, the ASMR group scored higher on Openness-to-Experience than did the Control group, *F*_(1, 559)_ = 91.939, *MSE* = 0.318, *p* < 0.001. Females also scored higher than Males on this trait, *F*_(1, 559)_ = 8.634, *p* = 0.003. These main effects were qualified by a significant interaction, *F*_(1, 559)_ = 8.515, *p* = 0.004. Despite the interaction, the difference between ASMR and Control groups was significant for both genders, with the effect being stronger for Females, *F*_(1, 559)_ = 85.41, *p* < 0.001, than Males, *F*_(1, 559)_ = 20.52, *p* < 0.001.

Also as predicted, the ASMR group scored higher than controls on Neuroticism, *F*_(1, 559)_ = 34.258, *MSE* = 0.630, *p* < 0.001. Females again scored higher than Males, *F*_(1, 559)_ = 35.845, *p* < 0.001. The interaction was not significant, *F*_(1, 559)_ = 1.827, *p* = 0.177.

Although not specifically predicted, the other personality dimensions also showed significant differences between the two groups. The ASMR group scored lower than Controls on Conscientiousness, *F*_(1, 559)_ = 61.576, *MSE* = 0.504, *p* < 0.001. Overall, females scored higher than males, *F*_(1, 559)_ = 4.833, *p* = 0.028; however, there was no interaction between the two factors, *F*_(1, 559)_ = 0.004, *p* = 0.947.

With respect to Extraversion, ASMR participants scored lower than Controls, *F*_(1, 559)_ = 41.165, *MSE* = 0.642, *p* < 0.001. There was no significant difference between Males and Females on this trait, *F*_(1, 559)_ = 2.409, *p* = 0.121. Although there was a significant interaction, *F*_(1, 559)_ = 8.159, *p* = 0.004, the difference between ASMR participants and Controls was significant for both Females, *F*_(1, 559)_ = 15.71, *p* < 0.001, and Males, *F*_(1, 559)_ = 55.61, *p* < 0.001.

The ASMR group also scored lower on Agreeableness than Controls, *F*_(1, 559)_ = 7.700, *MSE* = 0.483, *p* = 0.006, and Males scored lower than Females, *F*_(1, 559)_ = 8.985, *p* = 0.003. The interaction between Group and Gender was not significant, *F*_(1, 559)_ = 0.643, *p* = 0.423.

In summary, the predictions that ASMR participants would score higher on Openness-to-Experience and Neuroticism were confirmed. Although not specifically predicted, significant differences were also found on the remaining three personality dimensions, with ASMR participants scoring lower than Controls on Conscientiousness, Extraversion, and Agreeableness. The following analysis of the ASMR Checklist will determine whether similar relationships are found for ASMR participants differing with respect to the intensity of their ASMR experiences.

### The ASMR checklist

The ASMR Checklist, which was created for this study, aimed to both establish which types of stimuli tend to elicit the most intense ASMR experiences and to determine whether differences in the intensity of ASMR experiences are associated with higher or lower scores on the five dimensions of the BFI. To accomplish this goal, ASMR participants were asked to rate the intensity of ASMR experiences on a seven-point scale (from 0 to 6) for each of 16 stimuli. A large number of participants (*N* > 100) selected “unknown” for two of the Checklist items (“watching others sweep” and “watching others refill fountain pens”). These two items were subsequently dropped for the remainder of the analyses due to the large number of “unknown” responses. For the remaining 14 items, “unknown” responses were omitted from the analyses.

The stimuli were ranked from “most intense” to “least intense” as rated by the participants in this study. Due to the varying number of “Unknown” responses, the number of Valid responses (i.e., ratings from 0 to 6) were also calculated. The top ranked items (i.e., those with ratings above the mid-point of the seven-point scale) indicate the stimuli involving repetitive sounds were the strongest and most common triggers for ASMR experiences (see Table 2).

**Table 2:** **Descriptive statistics of intensity ratings for 14 stimuli that tend to trigger ASMR experiences**.

	***M***	***SD***	**Valid number**
Whispering	3.88	1.810	283
Haircut simulation	3.27	1.831	280
Tapping sounds	3.11	1.812	282
Scratching sounds	2.58	1.859	282
Watching someone touch another person's hair	2.52	2.022	261
Watching someone draw	2.41	1.911	257
Watching someone paint	2.41	1.956	251
Watching others apply makeup and/or nail polish to another person	2.02	1.814	244
Watching someone touch their own hair	1.84	1.806	258
Watching others open a package	1.81	1.811	262
Watching others apply makeup and/or nail polish to themselves	1.79	1.736	244
Dentist simulation	1.50	1.732	236
Chewing sounds	1.47	1.865	275
Watching others cook	1.39	1.632	241

The internal consistency of the ASMR checklist was evaluated using Cronbach's alpha. Based on the 173 ASMR participants (60.9% of ASMR participants) who rated all 14 stimuli on the checklist (i.e., did not indicate “Unknown” for any of the stimuli), Cronbach's alpha was.81, which indicates considerable agreement among the items. The items showing the strongest relationship with the total scale were “Watching Others Draw” and “Watching Others Apply Makeup and/or Nail Polish to Themselves.” The items showing the weakest agreement were “Chewing Sounds” and “Dentist Simulation.” Interestingly, the relatively low agreement for “Chewing Sounds” and “Dentist Simulation” is consistent with the results of (Barratt and Davis, 2015). In their survey study, “mouth sounds” tended to differentially affect individuals with ASMR by either eliciting the pleasant tingling sensations or sensations related to *misophonia* (hatred of specific sounds). It is therefore possible that the polarizing opinions reported by individuals with ASMR on mouth sounds affected the alpha value in the present experiment.

Mean intensity scores (*M*_Total_) were calculated for all 284 participants based on their responses to all items on the checklist (*M* = 2.35, *SD* = 1.01). The frequency distribution of *M*_Total_ corresponded to a normal distribution, as confirmed with a Kolmogorov-Smirnov test [*D*_(284)_ = 0.036, NS]. Due to the fact that *M*_Total_ could depend on the number of valid (i.e., not “Unknown”) responses, the total number of valid responses (Valid) was also calculated for each participant, (*M* = 12.87, *SD* = 2.04). Notably, the *M*_Total_ intensity score correlated negatively with the number of Valid responses, which means that average intensity was stronger for people responding to fewer items [*r*_(282)_ = −0.212, *p* < 0.01].

In addition to the calculation of an overall *M*_Total_ score, correlations among the 14 items and the reliability results suggested that somewhat distinct dimensions may underlie the checklist. Table 3 shows the five-factor results from a principal components factor analysis with Varimax rotation of the stimuli. Five factors had eigenvalues >1.0 and were retained. These five “intensity factors” were clearly defined by distinct items. Labels were chosen based on the items loading on each factor. These labels were Watching, Touching, Repetitive Sounds, Simulations, and Mouth Sounds, respectively.

**Table 3:** **Factor analysis of intensity ratings for 14 common triggers of ASMR experiences with accompanying intensity factor labels: watching, touching, repetitive sounds, simulations, and mouth sounds**.

	**1**	**2**	**3**	**4**	**5**
**Checklist item**	**Watching**	**Touching**	**Repetitive sounds**	**Simulations**	**Mouth sounds**
Watching others paint	**0.864**	0.259	−0.003	0.087	−0.060
Watching others draw	**0.844**	0.283	0.112	0.119	−0.079
Watching others open a package	**0.737**	0.099	0.158	−0.053	0.083
Watching others cook	**0.697**	0.142	0.006	0.010	0.198
Watching someone touch another person's hair	0.111	**0.847**	0.095	0.064	−0.046
Watching someone touch their own hair	0.168	**0.795**	0.160	−0.021	0.181
Watching others apply makeup and/or nail polish to themselves	0.472	**0.680**	0.030	0.070	0.053
Watching others apply makeup and/or nail polish to another person	0.344	**0.719**	−0.025	0.135	−0.044
Tapping sounds	0.125	0.049	**0.889**	0.097	0.034
Scratching sounds	0.065	0.130	**0.885**	0.099	0.038
Dentist simulation	0.007	−0.096	0.138	**0.869**	0.115
Haircut simulation	0.099	0.327	0.067	**0.775**	0.137
Chewing sounds	0.092	−0.173	0.006	0.093	**0.837**
Whispering	0.029	0.325	0.075	−0.105	**0.714**

*The bold values represent specific ASMR checklist items that load 0.60 or better on each factor*.

In summary, the ASMR checklist demonstrated internal consistency as measured by Cronbach's Alpha, and as well a possible multi-factor structure. The factor structure shown in Table 3 provides guidance in creating more items to tap into each factor.

### BFI scores and the ASMR checklist

The five personality dimensions were correlated with the *M*_Total_ and the five intensity factors: Watching, Touching, Repetitive Sounds, Simulations, and Mouth Sounds. The relationships for *M*_Total_ were based on the sample of 284 ASMR participants, whereas the correlations with the five factors were based on the subset of individuals who gave valid intensity ratings to each of the 14 stimuli (i.e., did not rate any stimuli as “Unknown”; *n* = 173). Positive correlations indicate that participants who tended to report more intense ASMR experiences on the checklist items scored *higher* on personality dimensions than participants reporting less intense reactions. In contrast, negative correlations indicate that participants who reported more intense ASMR experiences scored *lower* on personality dimensions.

Consistent with our predictions and with the fact that the ASMR group scored higher than Controls on Openness-to-Experience, *M*_Total_ intensity ratings correlated positively with Openness-to-Experience, *r*_(282)_ = 0.145, *p* = 0.014. The Openness-to-Experience effect appears to be primarily due to the Watching factor, *r*_(171)_ = 0.152, *p* = 0.047, and marginally with the Touching factor, *r* = 0.125, *p* = 0.101, whereas it was not correlated significantly with any other factor, |*r*|s < 0.08, *p*s > 0.35.

The correlation between Neuroticism and ASMR checklist scores was also consistent with our predictions and the earlier group analyses. The correlation between Neuroticism and *M*_Total_ was positive and significant, *r*_(282)_ = 0.117, *p* = 0.048, indicating that higher Neuroticism scores were associated with more intense ASMR experiences. The Neuroticism relationship was primarily due to the Touching factor, *r*_(171)_ = 0.188, *p* = 0.013, whereas none of its correlations with the other factors were significant, |*r*|s < 0.10, *p*s > 0.20.

We had no *a priori* predictions with respect to Conscientiousness, Extraversion, and Agreeableness. As such, our sole consideration was concerned with whether the correlations between *M*_Total_ and the five intensity factors agreed with differences observed between the ASMR and Control groups.

As the ASMR group scored lower on Conscientiousness than Controls, we expected this dimension to correlate negatively with the five intensity factors. Although it did not correlate significantly with the overall M_Total_, *r*_(282)_ = −0.023, *p* = 0.696, the correlation of Conscientiousness with the Repetitive Sounds factor was marginally significant, *r*_(171)_ = −0.137, *p* = 0.073. In other words, individuals who reported higher intensity responses to Repetitive Sounds had lower Conscientiousness scores. Correlations with any other factor were not significant, |*r*|s < 0.08, *p*s > 0.32.

The results for Extraversion and Agreeableness were not consistent with the observed group differences reported previously. Although the ASMR group scored lower than Controls on Extraversion, this dimension did not correlate significantly with *M*_Total_, *r*_(282)_ = 0.057, *p* = 0.340, or with any of the five intensity factors, |*r*|s < 0.12, *p*s > 0.12. Similarly, whereas the ASMR group scored lower than the Control group on Agreeableness, the correlation was positive and marginally significant for the overall *M*_Total_, *r*_(282)_ = 0.114, *p* = 0.055, significant for the Watching factor, *r*_(171)_ = 0.212, *p* = 0.005, and marginally significant for the Touching factor, *r*_(171)_ = 0.134, *p* = 0.079. Relationships with the other factors were not significant, |*r*|s < 0.08, *p*s > 0.32. This indicates that although individuals with ASMR were less agreeable than controls overall, *within the ASMR sample itself*, higher Agreeableness scores were correlated with greater intensity of tingling responses to stimuli that load in the Watching and Touching factors.

To summarize the intensity results, the correlations with Openness-to-Experience and Neuroticism were consistent with predictions and observed differences between the ASMR and Control groups, although modest in size. The negative correlation between Conscientiousness and one ASMR intensity factor was also consistent with our observed group differences. Correlations of intensity with Agreeableness and Extraversion were generally absent or in a direction opposite to the differences observed between groups. Given the preliminary nature of the checklist and its factors, these results require replication with the inclusion of additional variables that may be moderating the current results.

## Discussion

In the current study, we compared the five BFI personality scores (Openness-to-Experience, Conscientiousness, Extraversion, Agreeableness, and Neuroticism) of individuals with ASMR to control participants. We also conducted preliminary analyses to examine BFI personality scores as a function of intensity of ASMR triggers within the ASMR group. We first consider the primary predictions, which concerned the Openness-to-Experience and Neuroticism scales, and then briefly consider results for the other BFI dimensions.

### Predicted relationships

Previous research suggests that individuals who experience ASMR would score higher than controls on the Openness-to-Experience domain of the Big Five model of personality. This prediction was based on the assumption that ASMR participants would have increased sensitivity and receptivity to sensations. In addition, an enhanced sensitivity to aesthetic matters, as measured by this domain, could generalize to bodily sensations, such as the tingling experiences characteristic of ASMR. The results support this hypothesis as individuals with ASMR scored significantly higher on this domain than matched controls.

The reported higher scores of individuals with ASMR on this domain is also consistent with literature on the personality factors associated with frisson. Using physiological measures of frisson, Colver and El-Alayli (2016) found that frequency of frisson was positively correlated with participants' scores on the Openness-to-Experience domain of the Revised NEO Personality Inventory (NEO-PI-R; Costa and McCrae, 1992). The authors also reported that higher scores on Fantasy, Ideas, and Values (all cognitive sub-facets of Openness-to-Experience) were strongly and significantly correlated with frisson. Moreover, Grewe et al. (2007) found that those who focused more attention on music were more likely to experience frisson. As such, Colver and El-Alayli (2016) concluded that cognition should be emphasized when studying frisson. Because ASMR experiences also tend to arise when individuals focus on triggering stimuli, emphasizing the cognitive and attentional aspects of ASMR experiences may provide valuable insight into why some individuals experience ASMR. For example, past research has emphasized that trait levels of mindfulness may help to explain the unique sensory-emotional experiences reported by individuals with ASMR (e.g., Barratt and Davis, 2015; del Campo and Kehle, 2016).

Prior research by Barratt and Davis (2015) also led us to hypothesize that individuals with ASMR would differ from controls on the Neuroticism domain of the BFI. Barratt and Davis found a large proportion of moderate-to-severe levels of depression within their sample. Given that depression is associated with neuroticism, we expected individuals with ASMR to produce higher scores on Neuroticism than matched controls. This hypothesis was supported, as individuals with ASMR scored significantly higher than controls on Neuroticism, indicating lower levels of emotional stability.

Elevated Neuroticism scores for individuals with ASMR may be explained by increased self-consciousness, a sub-facet of Neuroticism, due to heightened awareness of physiological and/or psychological states during the ASMR experience. Consistent with this hypothesis, Rosmalen et al. (2007) found that Neuroticism correlated positively with a variety of somatic symptoms tied to internal bodily states. This may suggest that negative affect and ASMR share a common hyper-sensitivity to somatic and other interoceptive sensations.

On the Conscientiousness domain, individuals with ASMR scored significantly lower than Controls. However, the precise reason for this relationship is unclear. As there are no indications that individuals with ASMR are less self-disciplined than the general public, these results may instead be explained by more nuanced sub-facets of Conscientiousness, as opposed to the broad trait domain captured by the BFI. Future research is required to test this possibility.

The lower scores of ASMR participants on Extraversion could have a number of possible explanations. It may be that inward looking people are more likely to experience ASMR symptoms than more sociable, outward looking people. Alternatively, the ASMR symptoms may lead people to be less sociable and more introspective. That is, lower Extraversion (i.e., introversion) could contribute to the experience of ASMR symptoms or be a consequence of those symptoms. Additional research—preferably with behavioral and/or social manipulations—is necessary to tease apart these alternative explanations.

With respect to Agreeableness, the ASMR group scored lower than the Control group. It is not obvious why ASMR would be related to this dimension of personality and further research is clearly required. One approach would be to compare ASMR and control groups on the six facets that comprise Agreeableness. Identifying which facets are associated with the overall difference could shed light on the underlying processes.

### Psychometric properties of the ASMR checklist

The ASMR Checklist developed for this study demonstrated excellent reliability, indicating that diverse stimuli tend to produce reliable ASMR symptoms within individuals. This suggests some common underlying process by which a general sensitivity to stimuli elicits tingling sensations. This finding is also consistent with a possible relationship between ASMR and synesthesia, the tendency to experience sensations in a cross-modal or inter-modal manner, and implicates some common mediating process or pathway by which multiple types of sensation elicit a common response.

The ASMR Checklist also demonstrated that there is much variability across participants in ASMR triggers and their intensity. Indeed, factor analysis revealed that reported triggers clustered into five components rather than into a single “ASMR experience.” This finding suggests that there may be multiple ASMR subtypes, each involving a greater sensitivity to one or more types of triggering stimuli. The idea that ASMR can be a distinct, yet heterogeneous and highly individualized experience has been discussed in previous research (e.g., Barratt and Davis, 2015), and could be considered comparable to subtypes of other multi-modal perceptual experiences, such as synesthesia (e.g., grapheme-color synesthesia, chromestesia). For instance, an individual who is consistently triggered by soft whispering may not be triggered by soft chewing noises, while another individual is. Similarly, the perspective from which the stimulus is presented (i.e., first-person vs. third-person) may also differentially affect one's ASMR experience. It is also apparent that people differ in terms of the intensity of their ASMR tingles. The degree to which these characteristics—triggers and intensity—vary across the ASMR population will require further research with a substantial sample size. Such a study may also clarify whether these variables are related to how different people use their ASMR experiences (i.e., for the primary purpose of experiencing pleasant sensations or for the purpose of promoting relaxation).

Future studies involving task-based or resting-state functional magnetic resonance imaging would provide important corroborative evidence for the view that different ASMR subtypes exist. To date, the only neuroimaging study of ASMR involved an examination of the default mode network (Smith et al., 2016), one of many resting-state networks in the brain (Raichle, 2015). In this small study, individuals with ASMR showed reduced functional connectivity between regions of the default mode network. Activity within the default mode network also correlated in an atypical fashion with a number of additional brain areas, suggesting that resting-state networks that are relatively distinct in the general population are somewhat blended in ASMR. Future studies examining how the functional connectivity of different resting-state networks is affected by the sensitivity to different groups of ASMR triggers would provide biological evidence for the heterogeneity of this condition. Such analyses could be performed by adding scores from measures such as the ASMR Checklist as covariates when performing functional connectivity analyses.

Although the ASMR Checklist demonstrates much promise in furthering our understanding of this unique condition, no questionnaire can completely describe the atypical sensory-emotional associations of ASMR. One challenge in developing a checklist sensitive to different ASMR triggers lies in the fact that people differ in their exposure to various stimuli. For example, a common trigger for people with ASMR is watching another person draw, which is a stimulus that some individuals with ASMR may experience very infrequently. Further, there are additional common experiences that could be included in future versions of the checklist that would render it more universally applicable (e.g., cellular phone sounds). Future revisions of the scale based on empirical studies—and on input from the ASMR community—should help refine this instrument.

### Limitations and future directions

Although the Big Five Inventory developed by John et al. (1991) excels in its brevity and efficiency, it lacks in its ability to measure more nuanced sub-facets of personality (McCrae and John, 1992). For example, it is possible that the highly significant differences seen between ASMR participants and controls on the Neuroticism domain of the BFI may instead be due to significant differences in the self-consciousness sub-facet of Neuroticism rather than negative affect, which is the primary characteristic of this domain. Further, the differences seen on the Conscientiousness, Extraversion, and Agreeableness domains may be better explained by results obtained from a personality questionnaire that employs a faceted framework (e.g., Costa and McCrae, 1992). However, despite its limitations, the BFI is an excellent first step in identifying how the “Big Five” personality characteristics relate to ASMR, and provides a stepping-stone for additional research on this condition.

Future studies should also extend beyond the five personality traits examined in the current study. Indeed, the similarities between ASMR and flow states reported by Barratt and Davis (2015) suggest that trait mindfulness may be an interesting avenue of exploration. Additional studies could examine the potential therapeutic use of ASMR. The relaxation associated with this phenomenon would likely prove to be an effective remedy for stress and stress-related disorders.

Finally, there may be some inherent selection bias associated with our sample. The participants were likely regular-to-heavy internet users, as they were recruited through an online forum dedicated to discussions of ASMR triggers and experiences. A sample recruited from social media websites such as Reddit may be more willing to disclose information about their ASMR experiences and may be more naturally “Open to Experience” than the general population. As such, the sample at hand may not be representative of the overall ASMR population, especially those who do not share their experiences online. Alternative sampling methods could address this potential limitation and determine whether the findings with our select, albeit large, sample generalize to the entire ASMR population.

## Conclusion

The current study found reliable differences between ASMR and Control participants on five personality dimensions that may contribute to the condition. It also demonstrated the utility of a novel checklist that could help to identify processes and pathways that underlie the experience of ASMR symptoms. These results should therefore provide a foundation for future investigations into the causes and characteristics of this unusual sensory-emotional phenomenon.

## Author contributions

BF and SS designed the experiment. BF and Qualtrics Panels conducted the experiment. JC and BF analyzed the data. All authors contributed to and approved the manuscript.

## Funding

This research was supported by an Undergraduate Student Research Award (USRA) from the Natural Sciences and Engineering Research Council (NSERC) of Canada to the first author, and by an NSERC Discovery Grant and a University of Winnipeg Major Research Grant to the corresponding author. The authors gratefully acknowledge the University of Winnipeg Psychology Department for providing supplementary funding.

### Conflict of interest statement

The authors declare that the research was conducted in the absence of any commercial or financial relationships that could be construed as a potential conflict of interest.
