# Supplementary material for: An Examination of Personality Traits Associated with Autonomous Sensory Meridian Response (ASMR)
Source: Front Psychol. 2017 Feb 23;8:247. doi: 10.3389/fpsyg.2017.00247 (PMC5322228; doi:10.3389/fpsyg.2017.00247)
Supplement: Supplementary file 1 [file DataSheet1.docx]

Appendix A

ASMR Checklist

Please evaluate whether or not the following common stimuli trigger your ASMR, and if so, how intense the ASMR experience is on average when engaging with those stimuli.

**Intensity Scale (higher numbers represent increasing intensity):**

**0 = No Tingles**

**1**

**2**

**3 = Moderately Intense**

**4**

**5**

**6 = Most Intense ASMR Experience**

**or**

**Unknown**

Also, after perceiving a triggering stimulus, please estimate approximately how long it would take for this stimulus to cause tingles.

**For example, if whispering triggers the most intense tingles for someone, they would label that stimulus a "6". If it takes approximately 20 seconds after the whispering has started to trigger ASMR, this would also be indicated on the survey using the dropdown box.**

**** If you select "unknown" or "0" for stimulus intensity, leave the trigger time blank ****

| **Stimulus** | **Intensity (please circle the appropriate number according to the scale above OR select “Unknown”)** | **How many seconds after its onset do you feel tingles? (please circle one or select “Unknown”)** |
| --- | --- | --- |
| Whispering | 0 1 2 3 4 5 6  Unknown | 0 – 10 31 – 40  11 – 20 41 – 50  21 – 30 51 – 60 Greater than 60 Unknown |
| Tapping sounds | 0 1 2 3 4 5 6  Unknown | 0 – 10 31 – 40  11 – 20 41 – 50  21 – 30 51 – 60 Greater than 60 Unknown |
| Scratching sounds | 0 1 2 3 4 5 6  Unknown | 0 – 10 31 – 40  11 – 20 41 – 50  21 – 30 51 – 60 Greater than 60 Unknown |
| Chewing sounds | 0 1 2 3 4 5 6  Unknown | 0 – 10 31 – 40  11 – 20 41 – 50  21 – 30 51 – 60 Greater than 60 Unknown |
| Haircut simulation | 0 1 2 3 4 5 6  Unknown | 0 – 10 31 – 40  11 – 20 41 – 50  21 – 30 51 – 60 Greater than 60 Unknown |
| Dentist simulation | 0 1 2 3 4 5 6  Unknown | 0 – 10 31 – 40  11 – 20 41 – 50  21 – 30 51 – 60 Greater than 60 Unknown |
| [Optional] Other simulation, please specify if applicable: __________________________ | 0 1 2 3 4 5 6  Unknown | 0 – 10 31 – 40  11 – 20 41 – 50  21 – 30 51 – 60 Greater than 60 Unknown |
| Watching others open a package | 0 1 2 3 4 5 6  Unknown | 0 – 10 31 – 40  11 – 20 41 – 50  21 – 30 51 – 60 Greater than 60 Unknown |
| Watching others refill fountain pens | 0 1 2 3 4 5 6  Unknown | 0 – 10 31 – 40  11 – 20 41 – 50  21 – 30 51 – 60 Greater than 60 Unknown |

| Watching others apply makeup and/or nail polish to another person | 0 1 2 3 4 5 6  Unknown | 0 – 10 31 – 40  11 – 20 41 – 50  21 – 30 51 – 60 Greater than 60 Unknown |
| --- | --- | --- |
| Watching others apply makeup and/or nail polish to themselves | 0 1 2 3 4 5 6  Unknown | 0 – 10 31 – 40  11 – 20 41 – 50  21 – 30 51 – 60 Greater than 60 Unknown |
| Watching someone touch another person’s hair | 0 1 2 3 4 5 6  Unknown | 0 – 10 31 – 40  11 – 20 41 – 50  21 – 30 51 – 60 Greater than 60 Unknown |
| Watching someone touch their own hair | 0 1 2 3 4 5 6  Unknown | 0 – 10 31 – 40  11 – 20 41 – 50  21 – 30 51 – 60 Greater than 60 Unknown |
| Watching others draw | 0 1 2 3 4 5 6  Unknown | 0 – 10 31 – 40  11 – 20 41 – 50  21 – 30 51 – 60 Greater than 60 Unknown |
| Watching others paint | 0 1 2 3 4 5 6  Unknown | 0 – 10 31 – 40  11 – 20 41 – 50  21 – 30 51 – 60 Greater than 60 Unknown |
| Watching others cook | 0 1 2 3 4 5 6  Unknown | 0 – 10 31 – 40  11 – 20 41 – 50  21 – 30 51 – 60 Greater than 60 Unknown |

| Watching others sweep | 0 1 2 3 4 5 6  Unknown | 0 – 10 31 – 40  11 – 20 41 – 50  21 – 30 51 – 60 Greater than 60 Unknown |
| --- | --- | --- |
| [Optional] Watching any other mundane task, please specify if applicable: _________________________ | 0 1 2 3 4 5 6  Unknown | 0 – 10 31 – 40  11 – 20 41 – 50  21 – 30 51 – 60 Greater than 60 Unknown |
| [Optional] Any specific scent, if applicable: __________________________ | 0 1 2 3 4 5 6  Unknown | 0 – 10 31 – 40  11 – 20 41 – 50  21 – 30 51 – 60 Greater than 60 Unknown |
| [Optional] Any other notable trigger, please specify if applicable: ____________________ | 0 1 2 3 4 5 6  Unknown | 0 – 10 31 – 40  11 – 20 41 – 50  21 – 30 51 – 60 Greater than 60 Unknown |
| [Optional] Any other notable trigger, please specify: ____________________ | 0 1 2 3 4 5 6  Unknown | 0 – 10 31 – 40  11 – 20 41 – 50  21 – 30 51 – 60 Greater than 60 Unknown |
| [Optional] Any other notable trigger, please specify: ____________________ | 0 1 2 3 4 5 6  Unknown | 0 – 10 31 – 40  11 – 20 41 – 50  21 – 30 51 – 60 Greater than 60 Unknown |

**Are your experiences more intense when the stimulation is directed *toward you* (e.g., someone whispering to you)?** Y / N / Unsure

**Is your ASMR experience more intense when you observe someone performing an action to/for themselves (e.g., applying their own makeup) rather than when you observe someone acting upon another person (e.g., applying makeup to someone else)?** Y / N / Unsure

**How often do you use ASMR videos or audio files to help you go to sleep?**
a) Never
b) Less than Once a Month
c) Once a Month
d) 2 – 3 Times a Month
e) 2 – 3 Times a Week
f) Daily

**How often do you use ASMR videos or audio files to help you relax (but not sleep)?**
a) Never
b) Less than Once a Month
c) Once a Month
d) 2 – 3 Times a Month
e) 2 – 3 Times a Week
f) Daily

**Have you ever experienced “chills” from beautiful music?** Y / N / Cannot Recall

**If so, does the ASMR feeling differ getting chills from beautiful music?** Y / N

**If the ASMR feeling does *NOT* differ from experiencing chills from beautiful music, what genre(s) of music can cause ASMR?**

a) Pop
b) Country
c) Hip-Hop
d) Jazz
e) Classical
f) Choral Music
g) Other, please specify: ___________________

**On average, how pleasurable is an ASMR experience?**

a) Quite Uncomfortable
b) Mildly Uncomfortable
c) Neutral
d) Mildly pleasurable
e) Quite pleasurable

**What percent of the time is an ASMR experience *opposite* to how it feels on average? (For example, if ASMR generally feels pleasurable for you, what percent of the time does it feel uncomfortable?)**

a) 50 percent of the time.
b) Greater than 25 percent of the time, but less than 50 percent of the time.
c) Greater than 10 percent of the time, but less than 25 percent of the time.
d) Between 1 and 10 percent of the time.
e) ASMR virtually always feels the way I specified in the previous question.

**What is the earliest age you can recall having an ASMR experience? (For example, if you remember first having an ASMR experience at the age of four, please choose "4".**

**In this question, a "first ASMR experience" refers to the first time that you noticed you were experiencing ASMR-related tingles, even if at the time you did not know what they were called.**

**If you aren't sure, make your best guess.**

___ years old

**Have you ever had a seizure?**  Y / N / Unsure

**If yes, approximately how many have you had?** _____________________________
